# Progressive Medicine

**Published:** 1911-08

**Authors:** 


**Progressive Medicine,** Vol. 13, No. 2, June 1, 1911. **Lea and Febiger,** Philadelphia and New York. Three hundred and seventy-nine pages, illustrated. (Price, paper bound, $6.00; cloth, $9.00.)

The present number contains a discussion of hernia by Wm. B. Coley; of abdominal surgery by Arpad G. Gerster; of gynecology by John G. Clark; of diseases of the blood, diabetes, and the like, by Alfred Stengel; of Ophthalmology by Edward Jackson.

Being itself a digest of advances, discoveries and improvements, it is practically impossible to review this work thoroughly and we hesitate to select at random, any particular topic as a sample.

Progressive Medicine is by no means a short lived book, as the discussions, though based on current literature, are scholarly and thorough. We trust that some inventive genius will devise a binding material for this and similar works, more durable than thin paper and yet cheap enough to keep them within the reach of the mass of the profession.

